# HBV quasispecies composition in Lamivudine-failed chronic hepatitis B patients and its influence on virological response to Tenofovir-based rescue therapy

**DOI:** 10.1038/srep44742

**Published:** 2017-03-17

**Authors:** Priyanka Banerjee, Abhijit Chakraborty, Rajiv Kumar Mondal, Mousumi Khatun, Somenath Datta, Kausik Das, Pratap Pandit, Souvik Mukherjee, Soma Banerjee, Saurabh Ghosh, Saikat Chakrabarti, Abhijit Chowdhury, Simanti Datta

**Affiliations:** 1 Centre for Liver Research, School of Digestive and Liver Diseases, Institute of Post Graduate Medical Education and Research, Kolkata, India; 2 Structural Biology and Bioinformatics Division, CSIR-Indian Institute of Chemical Biology, Kolkata, India; 3 Department of Hepatology, School of Digestive & Liver Diseases, Institute of Post Graduate Medical Education and Research, Kolkata, India; 4 BioMedical Genomics Centre, National Institute of Biomedical Genomics, West Bengal, India; 5 Human Genetics Unit, Indian Statistical Institute, Kolkata, India

## Abstract

The present study sought to evaluate the structure of HBV quasispecies in Lamivudine (LMV)-failed chronic hepatitis B (CHB) patients and its impact in defining the subsequent virological responses to Tenofovir (TDF)-based rescue-therapy. By analyzing HBV clones encompassing reverse transcriptase (RT) and surface (S) region from LMV-failed and treatment-naïve CHB patients, we identified 5 classical and 12 novel substitutions in HBV/RT and 9 substitutions in immune-epitopes of HBV/S that were significantly associated with LMV failure. *In silico* analysis showed spatial proximity of some of the newly-identified, mutated RT residues to the RT catalytic centre while most S-substitutions caused alteration in epitope hydrophobicity. TDF administration resulted in virological response in 60% of LMV-failed patients at 24-week but non-response in 40% of patients even after 48-weeks. Significantly high frequencies of 6 S-substitutions and one novel RT-substitution, rtH124N with 6.5-fold-reduced susceptibility to TDF *in vitro*, were noted at baseline in TDF non-responders than responders. Follow-up studies depicted greater evolutionary drift of HBV quasispecies and significant decline in frequencies of 3 RT and 6 S-substitutions in responder-subgroup after 24-week TDF-therapy while most variants persisted in non-responders. Thus, we identified the HBV-RT/S variants that could potentially predict unfavorable response to LMV/TDF-therapy and impede immune-mediated viral clearance.

The advent of oral nucleoside/nucleotide analogs (NA) had ushered in a paradigm shift in the treatment of chronic hepatitis B (CHB) and currently represents the mainstay of HBV therapy. The NAs inhibit HBV replication by targeting the viral polymerase (Pol) that catalyzes the reverse transcription of a pre-genomic RNA intermediate to form mature, partially double-stranded, relaxed circular DNA genome of HBV^1^. However, complete eradication of HBV is not possible with the currently available NAs because of the intrinsic stability of the nuclear form of viral genome, the covalently closed circular (ccc) DNA and this necessitates the treatment to be continued for a long-term, if not indefinitely, which in turn is often associated with the development of antiviral resistance^1^. This risk is particularly high for NAs with low barriers to resistance and with overlapping resistance profiles. Antiviral resistance stems from the remarkable genetic variation of the virus that is inherently generated by low-fidelity HBV Pol during reverse transcription, leading to simultaneous presence of different but closely related viral variants (commonly defined as quasispecies) within the infected host and the subsequent selection of viral mutants, having a replication advantage in the setting of drug therapy^1^. Moreover, it is increasingly recognized that host immune responses are also relevant in the context of treatment induced clearance of chronic viral infection^2^. At present three nucleoside analogues [lamivudine (LMV), telbivudine and entecavir (ETV)] and two nucleotide analogues [adefovir (ADV) and tenofovir disoproxil fumarate (TDF)] have been approved for treatment of CHB, with LMV being the first agent made available^1^,^3^. However, LMV resistance develops in 53–76% of patients after 3 years of treatment^4^. Currently, ETV and TDF are recommended as first line therapies by major international guidelines^5^, but these relatively costly therapies are difficult to implement in countries having low or lower-middle income economies, such as India. Therefore, the less costly LMV, despite having low genetic barrier to resistance, is still extensively used resulting in a high risk of virological breakthrough and drug resistance and the management of the LMV resistant patients poses a growing problem for medical fraternity. Primary LMV resistance mutation has been mapped in the reverse transcriptase (RT) domain of HBV Pol and typically involved rtM204I/V, while compensatory mutations rtL180M, rtV173L, and rtL80I are often co-selected during therapy to restore HBV replication efficacy^1^. However, substitutions outside these well-defined RT positions had also been reported to be associated with LMV resistance^6^,^7^. Further, the Pol gene completely overlaps in a frame-shifted manner with the surface or envelope genes (preS1, preS2 and S) of HBV that contains both B- and T-cell antigenic epitopes, with the result that some drug-resistance mutations in Pol can directly impact the nature, function or antigenic properties of Hepatitis B surface antigen (HBsAg) that may have important immunological and clinical implications^1^. Different rescue therapies had been suggested for LMV resistance and typically involve switching to or increasingly, the addition of another antiviral agent such as ADV, ETV or TDF to the regimen^3^,^5^. Of these, TDF demonstrated a strong and lasting antiviral effect against wild-type HBV and has also been effective against LMV-resistant virus, thereby making it an attractive candidate for treatment of CHB patients who have failed LMV therapy^8^. It has been demonstrated that genetic heterogeneity of the virus at the level of quasispecies population represents an important determinant of viral persistence and response to therapy^9^. Hence, in this study we investigated the composition of HBV quasispecies in LMV-failed Indian patients and addressed the question of whether the extent of genetic variation of HBV bears any relationship to the response to TDF-based rescue therapy. We also studied the molecular evolution of the HBV quasispecies during the course of TDF-add on therapy by tracking individual viral variants in patients who exhibited different patterns of response to determine whether the viral quasispecies provides biological clues for understanding and predicting the outcome of rescue therapy in these patients.

## Results

### Distribution of HBV genotypes and demographic and clinical profiles of patients

A total of 37 CHB patients were enrolled in the study among which 18 had a history of failed LMV therapy [with a median duration of drug exposure being 34 months (range 27–42 months)] while 19 CHB did not receive any NA before their recruitment. The direct sequencing and phylogenetic analysis of S region of HBV derived from 18 LMV-failed patients revealed that 16 patients (88.9%) were infected with the HBV of genotype D (HBV/D), 1 patient (5.6%) carried HBV of genotype A (HBV/A) and 1 patient (5.6%) had HBV belonging to genotype C (HBV/C) (Supplementary Fig. S1A). Among the 19 treatment naïve CHB patients, 14 patients (73.7%) harbored HBV/D, 3 patients (15.8%) carried HBV/A and 2 (10.5%) were infected with HBV/C (Supplementary Fig. S1B). All subsequent analyses were done exclusively in patients carrying HBV of genotype D as they were the most representative group and included 16 LMV-failed and 14 treatment-naïve patients. The baseline clinical and demographic profiles of the two groups of patients with HBV/D showed no significant difference in viral/biochemical/clinical parameters at point of their enrollment in the study (Supplementary Table S1).

### Analysis of RT domain of HBV Pol in the LMV-failed and treatment-naïve CHB patients

Direct sequencing of the PCR amplified RT domain of HBV/D from 16 LMV-failed patients demonstrated the presence of well-known LMV resistance mutations, rtM204V/I, rtL180M, rtS202G, rtL80I and rtA181T, either singly or in combinations. Notably, rtM204V/I were found in 12 out of 16 (75%) LMV-failed patients, of which 9 (75%) had rtM204V in association with rtL180M while the other 3 (25%) patients had rtM204I together with rtL80I mutation. In the remaining 4 (25%) patients, the multi-drug resistant mutation rtA181T was identified. Out of the 9 patients whose HBV harbored rtM204V mutation, four patients also had rtS202G in their HBV DNA. None of the above-mentioned LMV resistant mutations were detected in HBV from treatment-naïve patients.

Further, to gain an insight into the composition of HBV quasispecies in patients who were non-responsive to LMV therapy, we adapted a cloning-sequencing approach whereby sequences of RT/S region of 10 HBV clones from each of 16 HBV/D infected patients were analyzed and compared with analogous sequences of similar number of clones from each of 14 HBV/D infected NA-naïve patients. For those LMV- failed patients whose HBV carried either rtM204V + rtL180M or rtM204I + rtL80I substitutions, these viral variants were found to comprise 20–100% or 50–100% respectively of said viral quasispecies pool. The rtA181T variant was detected in 10–40% of the HBV clones from four LMV-failed patients while the clonal prevalence of rtS202G ranged from 20–100%. Interestingly, the clonal analysis indicated that a total of 19 amino acid (aa) substitutions were present in significantly higher frequencies at the nominal level of 0.05 in the LMV-failed CHB patients as compared to untreated group (Table 1). These include five classical LMV-resistant mutations, rtM204V, rtM204I, rtL180M, rtS202G and rtL80I and 14 novel substitutions namely, rtN53D, rtY54H, rtL91I, rtI121L, rtF/H122L, rtH124N, rtQ130P, rtN131D, rtA219S, rtH248N, rtS256C, rtR266I, rtH/L267Q and rtV278I. However, in order to account for multiple comparisons, the Benjamini-Hochberg adjustment was applied to significance calculation following which, the difference in frequencies of 17 out of 19 rt variants (with the exception of rt54H and rt256C) remained statistically significant between the two groups (Table 1). Remarkably, HBV clones having the mutational combination of rtM204V + rtL180M always carried the novel substitution rtH/L267Q while those with rtM204I + rtL80I co-existed with rtR266I. Since the significant difference in the frequency of mutations in HBV clones from the two groups of patients could be attributed to a few individuals carrying a specific mutation in majority of its clones, we also investigated whether the proportion of individuals harboring a specific mutation in any of its HBV clones is significantly different in the two groups. Out of the 17 aa substitutions where significant differences were observed in frequencies between HBV clones, 10 substitutions (rtN53D, rtI121L, rtF/H122L, rtH124N, rtQ130P, rtN131D, rtL180M, rtM204V, rtM204I and rtV278I) exhibited significant differences in the proportion of individuals having HBV clones carrying the corresponding mutation.

### Mapping of LMV associated mutations in HBV RT domains and co-occurrence of mutations

**Figure 1:**
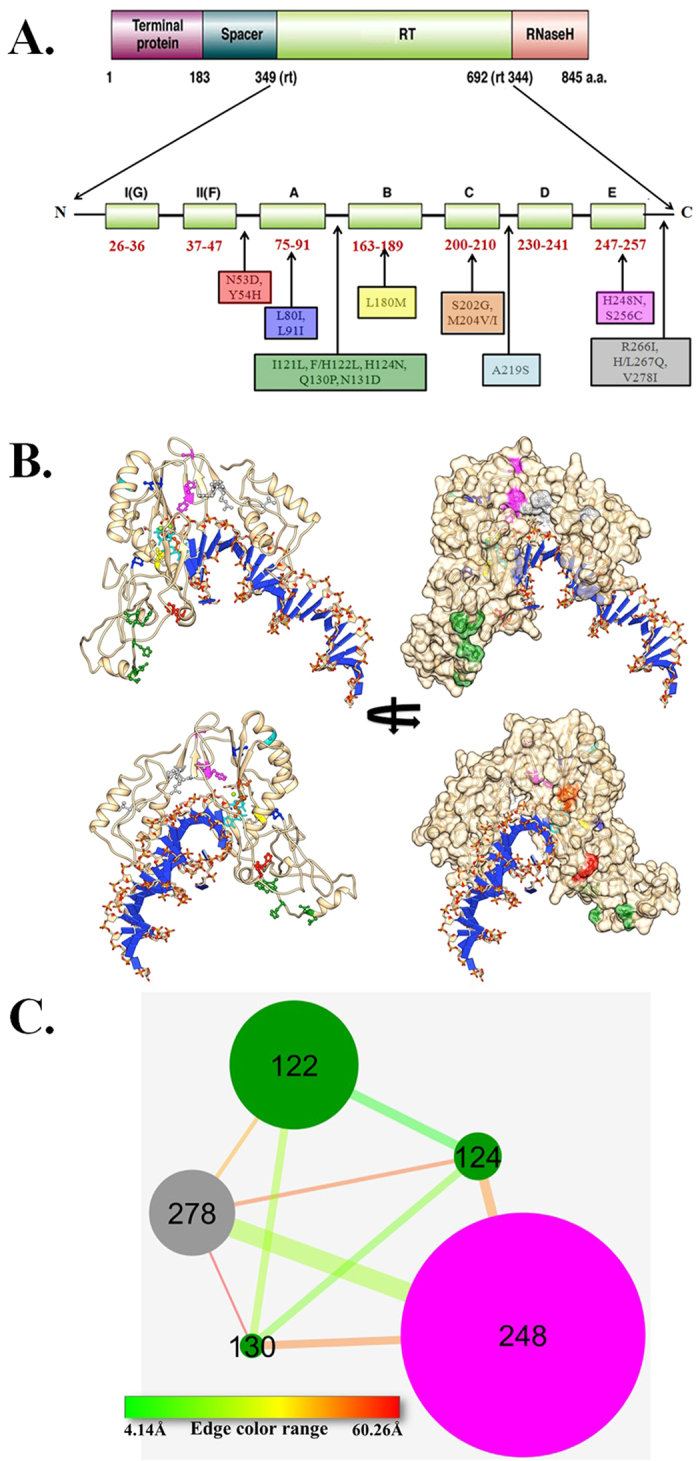
Mutational mapping of HBV RT domain. Panel A maps the observed point mutations onto the two-dimensional (2D) domain architecture whereas Panel B shows the mapping of the same mutations onto the 3D model of HBV RT domain. Same color code is maintained for the mutation cluster depicted in panel A and B, respectively. Bound substrates (TTP and DNA) are shown in stick model (cyan and blue, respectively). Panel C provides the network of most frequently co-varying mutation pair sites within the HBV strains where two co-mutating sites are connected via edges. Edge width depicts the frequency of each mutation pair whereas the color of the edge represents the inter-residue distance of the co-varying positions. Node size is inversely proportional to their distance from the active center. Node color is maintained as Panel A and B.

We next analyzed the distribution of LMV-related aa substitutions identified by clonal analysis in seven functional domains (A–G) and six interdomains of HBV RT. Six (rtL80I, rtL91I, rtL180M, rtS202G, rtM204V/I, rtH248N) (35.3%) out of 17 substitutions were located within different RT domains while 11 substitutions (64.7%) were mapped in inter-domains. The maximum number of aa substitutions (5 out of 11; 45.5%) were detected in the A–B inter-domain of RT, which overlaps with the “a”-determinant region of HBV surface protein (aa s124–147). While two-dimensional (2D) mapping indicates region specific clustering of frequently mutated residues (Fig. 1A), mapping of the same on the three-dimensional (3D) model of HBV RT domain generated in the study (Fig. 1B), showed the spatial proximity of certain sites despite their sequential distances. Several substitutions occurring at sites rtL80 and rtL91 (color: blue), rtL180 (yellow), rtS202 and rtM204 (orange), rtH248 and rtS256 (purple), rtR266 and rtH267 (grey) were found to be reasonably close to the active center of the RT domain. However, few mutation sites, such as rtN53 and rtY54 (red), rtI121, rt122, rtH124, rtQ130 and rtN131 (green), rtA219 (cyan) and rtV278 (grey) were relatively distant with respect to the active site residues. Mutations at sites proximal to the active center could directly modulate the architecture of the substrate and/or substrate analogous inhibitor/drug binding pocket whereas distal mutations could impact the binding via allosteric modulations. However, at this stage, the suggestion of distal modulation is speculative and needs to be testified via rigorous molecular dynamics and/or relevant biophysical studies. Interestingly, several point mutations were found to be co-occurring with mutation at another site indicating a correlated mutational behavior of the HBV strains in order to attain drug resistance. The most frequently observed co-mutations in the current study are listed in Supplementary Table S2 whereas Fig. 1C presents a network of such co-varying positions along with their occurrence frequency and proximity to the active center. It is interesting to notice that active center closest site rtH248 was co-mutating most frequently with an apparent distal position, rtV278. Similarly, a group of distal mutation sites rtF122, rtH124 and rtQ130 (green) were not only co-varying together but also were correlated to mutations occurring at rtH248 and rtV278. These co-mutational patterns between proximal and distal sites further suggest the existence of long-range structural and functional modulation of HBV RT domain with respect to mutations.

### Analysis of the small S gene in the HBV quasi species pool isolated from the LMV failed CHB

Owing to the overlap of RT region of HBV Pol with the S gene coding for HBsAg, we also investigated the HBsAg mutations that were selected during long-term LMV therapy and correlated with treatment failure. HBsAg, having a complex trans-membrane topology, represents a key target of host antiviral immune responses and contains recognition sites (epitopes) for B cells as well as cytotoxic T (Tc), helper T (Th) cells. The clonal analysis uncovered 20 amino acid substitutions in ORF-S that showed nominally significant difference in frequencies between LMV-failed and untreated patients. However, out of these 20 S-substitutions, 10 (sT114S, sT126I, sP127T, sN131T, sN143S, sY/S161F, sA194V, sI195M, sW196L and sR210N) remained significant after application of Benjamini-Hochberg correction (Table 2). Among these, sT114S, sA194V and sW196L corresponded to rtF/H122L, rtS202G and rtM204V respectively. Notably, 9 out of the 10 substitutions resided in the known B- and T-cell epitopes of HBsAg that included 4 substitutions (sT126I, sP127T, sN131T and sN143S) within the “a”-determinant region (aa s124–147), the primary target for neutralizing antibody produced by B cells during natural infection or following active or passive immunization^10^.

### Hydrophobicity plot

**Figure 2:**
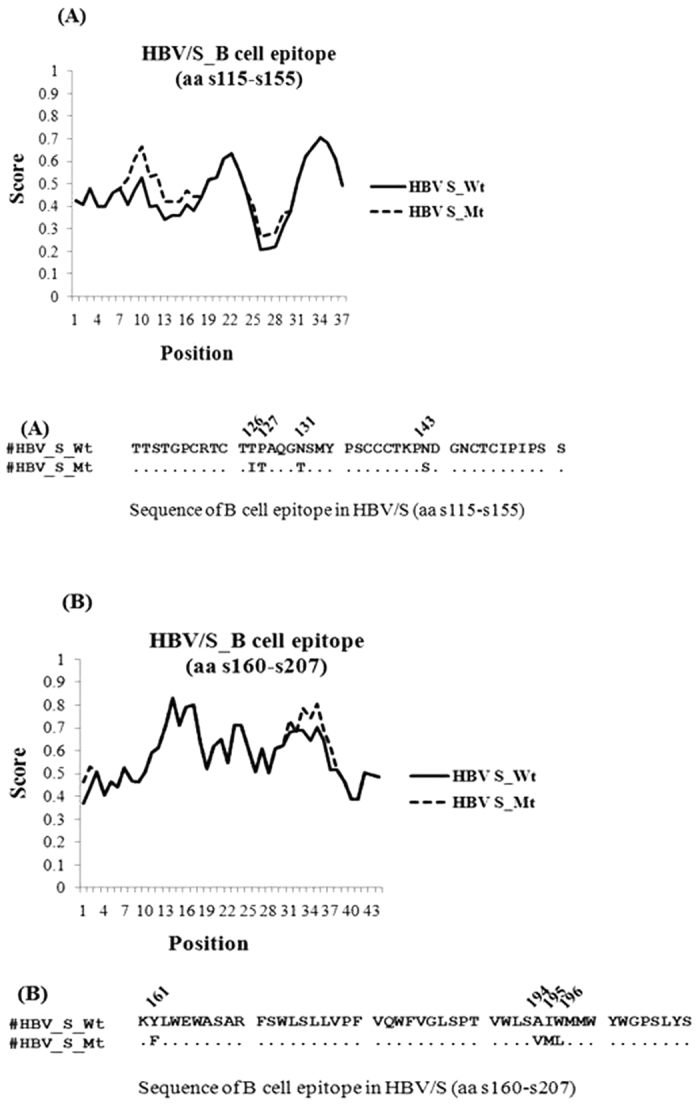
Hydrophobicity plots of B-cell epitope regions- (**A**) aa s115–155 and (**B**) aa s160–207 of HBV/S showing altered hydrophobicity due to substitutions at specific positions, obtained by the Kyte and Doolittle method using PROTSCALE program in EXPASY. Values were normalized between 0 and 1, and a sliding window of five amino acids with a step size of 1 was applied.

We examined the impact of the amino acid substitutions within the B-cell epitopes (aa s115–155 and s160–207) of HBAg on the hydrophobicity profile of the specific epitope regions by the Kyte–Doolittle method^11^. Most of the substitutions in B-cell epitopes resulted in variations in epitope hydrophobicities relative to wild-type residues (Fig. 2A,B), with the exception of sP127T, where no difference was noted. A similar change in hydrophobicity profiles due to these substitutions in the overlapping T-cell epitopes was noticed. Thus it seems plausible that these mutated B- and T- cell epitopes of HBsAg could contribute to viral immune escape and establishment of HBV persistence in LMV-failed patients.

### Therapeutic regimen and clinical course of the LMV-Failed CHB patients

In this study, all 16 HBV/D infected, LMV-failed patients were treated by adding TDF (300 mg/day) in their LMV-based regime and followed for one year. However, 6 patients were excluded due to poor compliance to therapy. Of the 10 patients who completed 48 week follow-up, 6 patients exhibited greater than 1 Log10 copies/ml reduction in HBV-DNA after 24 weeks of therapy and hence considered to be responders to TDF add-on therapy (Supplementary Fig. S2). Continuation of therapy thereafter resulted in a further yet small reduction in viral load of 0.7 Log10 at 1 year in these patients although none could achieve undetectable HBV-DNA. However, in the remaining 4 patients, the addition of TDF did not result in any significant change in viral load and transaminase values between initiation of TDF therapy and at 24 or 48 weeks and hence considered as non-responders.

### Baseline Clinical and demographic profiles of the LMV experienced CHB patients with differential response to add-on TDF therapy

The clinical and virological profiles of all 10 LMV-failed patients who completed 48-week TDF therapy were assessed at baseline and no significant differences were seen in age, sex and serum ALT levels between the responders (n = 6) and non-responders (n = 4). However, the responders exhibited higher mean HBV-DNA level of 6.526 ± 0.4680 log10 copies/ml than that of the non-responders with 3.818 ± 0.3460 Log10 copies/ml (Supplementary Table S3). The percentage of patients with HBeAg negative status was higher in nonresponders (2 out of 4 patients, 50%) than that in the responders (2 out of 6 patients, 33.33%).

### HBV RT/S quasi species heterogeneity at Baseline in LMV resistant patients with different therapeutic responses to TDF

To ascertain whether the response to TDF add-on therapy correlates with the extent of baseline genetic heterogeneity of HBV/D isolates among LMV resistant patients, we assessed the genetic diversity and complexity of HBV quasispecies in the responders and non-responders. At baseline, the quasipecies complexity at the amino acid level was found to be significantly low in TDF-responders than non-responders (0.7500 vs 0.9300, p < 0.05), although the viral complexity at nucleotide level, the mean genetic distance (d), the number of synonymous substitutions per synonymous site (dS), and the number of non-synonymous substitutions per non-synonymous site (dN) did not differ statistically between the two groups (Supplementary Table S4).

We separately screened the RT and S regions of viral population in LMV-failed patients to identify specific amino acid differences that might discriminate TDF-responders from non-responders. The clonal analysis showed that there was no significant difference at baseline in the frequencies of classical LMV-resistant mutations, rtL80I, rtL180M, rtS202G and rtM204V/I in both groups of patients. However, the frequencies of 12 novel amino acid substitutions in RT and 8 substitutions in HBV-S region were found to be significantly different at a nominal level of 0.05 between TDF responders and non-responders at baseline (prior to the initiation of TDF-based rescue therapy) (Table 3). Among these, only one RT-variant, rtH124N and 6 S-variants sS45A, sA/S113T, sT118V, sT125M, sA128V and sR210N remained significant following Benjamini Hochberg adjustment (Table 3), implying that the presence of these variants were associated with non-response to TDF. Except for A/S113T, all the other 5 S-substitutions were localized in B- or T-cell epitope regions.

### Susceptibility of rtH124N mutant and wild-type HBV to Tenofovir *in vitro*

To determine whether rtH124N mutation impact the viral susceptibility to tenofovir, we engineered rtH124N into wild-type (WT) laboratory strain of HBV belonging to genotype D and tested the abilities of WT and rtH124N mutant HBV to replicate in Huh7 human hepatoma cells in the presence of increasing concentrations of tenofovir by quantifying intracellular HBV DNA levels by real time PCR. For WT, 6.7 ± 0.4 μM tenofovir reduced the replicative level to 50% while the presence of rtH124N increased the IC_50_ to 44.0 ± 3.2 μM, that corresponded to a 6.5 fold decreased susceptibility to tenofovir (Supplementary Fig. S3).

### Examination of clonal evolution of HBV during the course of TDF add-on therapy

We investigated the pattern of quasispecies evolution at 24 and 48 week following the initiation of TDF-therapy by characterization of viral population on serial samples from six responders and four non-responders. While the responder group displayed a dramatic reduction in viral complexity and diversity including, Shannon entropy at nucleotide and amino acid level, d at nucleotide and amino acid level, dS and dN with respect to baseline following 24 and 48 weeks of TDF therapy, the non-responders were typified by less pronounced alteration of viral quasispecies during the same period, suggesting that preexisting variants were mostly maintained with possible changes in their proportion (Supplementary Table S5). We further appraised the changes in individual amino acid residues after 24 and 48 week TDF add-on therapy that might impact the therapeutic response. Interestingly, there was no statistically significant difference with respect to baseline in the proportions of LMV-associated mutations, rtM204V, rtL180M and rtS202G in both TDF responders and non-responders even after 48 week-therapy. However, at the end of 24-week of TDF therapy, decline in the frequencies of rtM204I, rtL80I, rtN53D, rtY54H, rtH124N, rtN131D and rtH248N together with sS45A, sP46T, sI68T/A, sA/S113T, sT118V, sT125M, sW196L and sR210N from basal values were noted exclusively in the TDF-responder group at the nominal 0.05 significance level (Table 4). After implementing Benjamini-Hochberg correction, 3 (rtM204I, rtL80I, rtY54H) out these 7 RT substitutions and 6 (sS45A, sP46T, sI68T/A, sT118V, sW196L and sR210N) out of 8 S-substitutions remained significantly less frequent in the responders (Table 4). At 48 week of TDF therapy, the frequencies of rtM204I, rtL80I, sS45A, sT118V, sW196L and sA128V continued to be significantly reduced from baseline in responders, although an increase in frequency of the substitution rtV278I was noted in the HBV clones of the responders during this observational period (Table 5). In contrast, there were no significant change in percentages of RT and S variants from the baseline values in the non-responders after 24 and 48-week TDF therapy following Bejamini-Hochberg adjustment, although the frequencies of rtM204I and rtL80I were found to be nominally significantly lower than baseline frequency post 24 week and 48 week therapy (Tables 4 and 5).

## Discussion

The burden of historical and continued LMV use in India is very large and its widespread use favors the selection of HBV variants that not only confer resistance to this drug and exacerbations of liver disease but could potentially affect the success of rescue therapy with another anti-HBV agent. Analysis of the viral population has become increasingly important in the clinical management of HBV patients, both in the initial design of a therapeutic plan and in selecting a salvage regimen. In the present study, we first investigated the composition of HBV quasispecies in NA-naive and LMV-failed CHB patients of Eastern India with matched genotype to get a snapshot of the variability of overlapping RT/S region of HBV arising due to extended LMV therapy. Our mutational analysis focusing on the RT domain of HBV Pol revealed that 75% of our cohort of HBV/D infected patients, who were under LMV for a median duration of 34 months carried the classical rtM204V/I mutation, of which rtM204V was predominant. A previous study from North India reported the presence of rtM204V/I resistant mutants in 6% and 29% CHB patients receiving LMV for 12 and 18 months respectively^12^ while another study from South India detected M204V/I mutations in 27% of LMV-treated patients after a median treatment period of 13 months^13^.

The most widely used method of detecting drug-resistant HBV in majority of the studies involves PCR amplification and direct sequencing of the RT region and though informative, this method could detect only the highly abundant variants. Hence to achieve a more precise characterization of the ensemble of variants that compose the HBV population in the enrolled patients, we adapted a cloning-sequencing approach, whereby a panel of 10 HBV/D-RT clones was generated such that each clone represented an independent variant within the quasispecies. The analysis of HBV clones from LMV-failed and untreated CHB patients expanded the spectrum of LMV-associated mutations to include 12 novel RT substitutions in addition to the 5 well-known ones. Our 3D homology model of RT showed a close proximity of some of frequently mutated sites, such as rtL80 and rtL91, rtH248, rtR266 and rtH267 to the active center of the RT domain demonstrating their possible roles in the binding of the nucleotides and inhibitors and in the development of resistance. Further, we noted that many RT positions co-mutate with more than one other position, suggesting that these correlated mutations are more likely to be associated with drug resistance than expected by chance. Among the non-classical RT substitutions detected in the study, rtL91I was earlier noted in HBV of genotype D, rtH124N and rtV278I were perceived in HBV of genotype G while rtN131D was encountered in HBV of genotype B derived from different CHB patients under LMV monotherapy in USA^6^. In two different studies, Ciancio *et al*.^14^ reported the association of rtC256 and rt91L/I with failure of long-term response to LMV while Wong *et al*.^15^ suggested rt124N to be associated with suboptimal response to ETV.

A series of 10 S-gene substitutions within different immune epitopes were seen to emerge in the viral population of patients failing LMV therapy. Of these, sI195M and sW196L had been shown to have reduced binding to anti-HBsAg antibodies^16^ while a decrease in the antigenicity of the S protein due to the presence of sP127T mutations had been documented^17^. It is widely recognized that the humoral immune responses to HBV surface proteins contributes to the clearance of circulating HBV particles, whereas cellular immune responses are responsible for the elimination of infected hepatocytes^18^. Our analysis of epitope regions with and without the identified substitutions showed changes in their hydrophobicity profiles, suggesting that these substitutions may affect the recognition of viral immune targets by specific B- or T-cells. There is increasing evidence that the success of anti-viral therapy is conditioned by contribution of host immune system^19^ and it appears that extended LMV therapy also favored the selection of HBV with deep modification in S protein that could accentuate the ability of the variant virus to evade the host immunity and persist in hosts. Thus our study confirms that LMV-failed Indian patients carried a constellation of recognized and novel mutations in RT and in immune epitopes of S region of HBV/D that undermine the efficacy of LMV and pose a serious challenge for the management of these patients.

Clinical evidences suggest TDF monotherapy or TDF plus LMV combination therapy to be a favorable therapeutic option in CHB patients infected with LMV-resistant HBV^20^. In a previous study, in HIV co-infected patients with LMV-resistant HBV infection, the use of TDF + LMV combination therapy was found to be effective at reducing the HBV-DNA to undetectable level in 76% of the patients during a median treatment period of 116 weeks^8^. An even better response rate to TDF + LMV therapy in LMV-resistant patients was reported from a multi-centre study in Korea, where 92.6% of the LMV-resistant patients achieved <20 IU/ml of HBV DNA load after ~3 months of combination therapy^8^. Another recent study depicted that 85.4% of Korean CHB patients with LMV and ETV resistance achieved virologic response (HBV DNA level <20 IU/mL) following 24 months of TDF mono-rescue therapy^21^. The salvage potential of TDF was also evaluated in HBV genotype C infected Japanese patients who had failed treatment with other NAs. Serum HBV-DNA levels were found to be undetectable in 59% of subjects at week 24, and increased to 62% and 71% at weeks 48 and 96, respectively^22^. However, another study from Italy documented that in LMV-failed patients with a median treatment duration of 19 months, LMV + TDF therapy resulted in HBV-DNA undetectibility (<2,000 copies/ml) in 20% of patients at 24 months^23^. In the present study, 60% of the LMV-failed patients who received TDF add-on therapy showed >1 log reduction in viral load from their baseline at the end of 24 week although none achieved undetectable HBV-DNA even after 48 week therapy. A possible explanation of the lower rate of virological response in our patients is their extensive prior exposure to LMV (median duration 34 months) that had induced the emergence of a highly heterogeneous quasispecies pool and the reduced sensitivity of some of the viral variants to TDF.

Different studies have sought to predict the efficacy of antiviral therapy based on viral quasispecies parameters (complexity and diversity) before or during antiviral therapy and often yielded conflicting results. Chen *et al*. and Liu *et al*. investigated the changes of HBV quasispecies in the RT region in CHB patients who received LMV or ETV treatment, respectively^9^,^24^. In both studies, HBV quasispecies variability at baseline was found to be comparable in responders and non-/or partial responders but higher changes in complexity and diversity of HBV quasispecies in the first 4 weeks of therapy correlated with a good antiviral efficacy. However, in another study, Wong *et al*. reported that a lower pre-treatment or baseline quasispecies complexity and diversity was associated with poor response to ETV^15^. Further, a more recent study showed that the evolution of HBV quasispecies was not significantly different in patients with incomplete ADV response during TDF-based therapy, whether they were slow or rapid responders^25^. In LMV-failed patients subjected to TDF add-on therapy, we also conducted analysis of genetic parameters of HBV on a subset of TDF-responders and nonresponders using serum samples obtained at baseline and while on treatment. It was observed that a greater level of amino acid complexity in the RT/S region at baseline was significantly associated with a higher likelihood of nonresponse to TDF. Further, comparison of the genetic heterogeneity of the baseline samples versus the composite of follow-up samples revealed that the pattern of quasispecies evolution differed significantly between responding and non-responding patients, whereby the responders experienced a striking reduction in genetic diversity leading to an increasingly homogeneous viral population while there was a lack of progressive shift in the viral population in nonresponders. Intriguingly, in-depth analysis of the baseline quasispecies pool showed that while both TDF responders and non-responders carried the well- known LMV resistance mutations in comparable proportions, there were significant differences in the basal frequencies of the one novel RT (rtH124N) and 6 S-substitutions (sS45A, sA/S113T, sT118V, sT125M, sA128V and sR210N) between the two groups. Thus, these substitutions could be considered as significant predictors of response to TDF-based rescue therapy for LMV-failed patients as the increasing presence of these variants could hinder the response to therapy as well as immune clearance of the virus. We assessed the susceptibility of rtH124N to tenofovir in transiently transfected Huh7 cells *in vitro* and noted that HBV harboring rt124N consistently demonstrated a 6.5-fold increase in the IC_50_ values as compared to WT-HBV. By definition, a 5- to 10-fold increase in the IC_50_ correlates with partial resistance^26^ and hence our data clearly demonstrated partial resistance to tenofovir in the presence of rtH124N polymerase mutation and also strongly indicate the association of rt124N with a slower response to TDF therapy. With respect to the substitutions in immune-epitopes of HBV/S, the immune response originally elicited by specific epitopes or their mutated forms need to be carefully dissected to understand the dynamics of viral escape from a co-evolving immune response during therapy.

Following TDF therapy, the frequencies of rtM204I and rtL80I were found to decrease in both responders and non-responders although there was little change in the prevalence of rtM204V and rtL180M. *In vitro* studies conducted separately by Lada *et al*.^27^ and Delaney *et al*.^28^ documented that HBV bearing the rtM204V + rtL180M mutations displayed a 3.33 and 2.1-fold increase in the IC_50_ for TDF compared to wild-type virus in cell culture suggesting that TDF was less efficacious on these LMV-resistant mutants. Our results further extended these previous findings and raised doubts about the effectiveness of TDF on the heavily LMV experienced Indian patients, where rtM204V + rtL180M mutation is widely prevalent. We noted that the baseline frequency of rtM204V (18.3%) substitution was less than that of rtM204I (25%) in the clonal population of the LMV-failed patients who responded to TDF therapy in contrast to the non-responders where rtM204V was dominant. Given that none of the TDF responders could achieve undetectable HBV DNA levels even after 1 year of therapy, it appears that the persistence of the novel RT variants in the quasispecies pool (such as, rtV278I) might cause a reduced sensitivity to TDF and could undermine the therapeutic benefit. Hence a longer duration and a higher dose of TDF therapy may be required for these patients to reach undetectable HBV-DNA levels. Additionally, HBV population in TDF responders showed a significant decline in the frequency of S-substitutions from baseline during the course of therapy, probably reflecting in at least partial restoration of immune responses in parallel with reduction of viral load. In contrast, the HBV-associated immune dysfunction continued to persist in TDF non-responders where there was little change in viral load and the number of S-substitutions before and after the add-on therapy. Clearly the role played by the immune response in the setting of anti-viral therapy is complex. While the drugs provide a constant selection pressure, the immune response is sensitive to changes in the antigenic structure of the virus population^29^ and also may shift between different viral epitopes^30^. A comprehensive evaluation of the immune response dynamics during the course of TDF therapy in CHB patients is needed to better understand the outcome of therapy.

Finally, through extensive characterization of viral quasispecies in LMV-failed patients and their evolution during add-on TDF therapy, we identified HBV sequence patterns in RT and S that have the potential to serve as predictors of long-term drug response to LMV and TDF. This knowledge could help in the development of novel strategies engaging immune-based and anti-viral selection synergistically against HBV. However the potential limitations of the present study lie in the small number of patients and analysis of only 10 clones per time point. Nonetheless our findings reiterated the importance of carrying out targeted surveillance of viral population of the patients before initiation of any therapy. The ability to distinguish between individuals who are likely to benefit from therapy from those who have little chance of long-term sustained response could significantly improve the utility and reduce the overall treatment costs and additional side effects of these anti-HBV treatment regimes.

## Materials and Methods

### Patients

This was a prospective study that recruited LMV-failed CHB patients from School of Digestive and Liver Diseases (SDLD), Institute of Post Graduate Medical Education and Research (I.P.G.M.E. & R.), Kolkata, India during 2010–2013. Adult patients who were under uninterrupted LMV monotherapy for minimum 24 months but showed insufficient virological response to LMV, defined as a reduction in HBV-DNA of <1 log copies/ml or a persistent viraemia (HBV-DNA levels >10^4^ copies/ml) and elevated alanine transaminase (ALT) (>40 IU/ml) since the preceding 6 months or more of their visit to SDLD were considered for the study. Patients were excluded if they had previously used any anti-HBV agent, other than LMV, or had coexisting HCV, HDV or HIV infection, decompensated liver disease or evidence of HCC. In addition, treatment-naïve CHB patients having viral load >10^4^ copies/ml, ALT > 40 IU/L and without any other viral infection were included as a comparison group.

### Rescue therapy and follow-up after enrolment

Patients with LMV failure, who satisfied the inclusion and exclusion criteria were given TDF (300 mg) “add-on” LMV (100 mg) daily for at least one year and their clinical and virological status were reviewed at 24 and 48 weeks.

### Collection of blood samples

On the day of enrolment, 5 ml blood was collected from LMV-failed as well as treatment-naïve patients, the serum was separated and stored at −80 °C refrigerator until use. Subsequently, blood samples were collected from LMV-failed patients at 24 week and 48 week following commencement of TDF add-on therapy. Compliance with treatment was assessed by interview during every visit. Written informed consents were obtained from all patients and the access to human samples and all experimental protocols were carried out in accordance with the approved guidelines of the Ethical Review Committee of I.P.G.M.E.&R.

### Testing of liver enzymes

Each serum sample was tested for the levels of liver enzymes, ALT and aspartate aminotransferase (AST) using commercially available kits.

### Serum HBV DNA Isolation and assessment of Viral Load

HBV DNA was extracted from 200 μl serum samples using Blood Mini Kit (Qiagen Inc., CA, USA). At baseline and each follow-up point, the quantification of HBV DNA was performed by Real-Time PCR using SYBR Green Master mix (Applied Biosystems [ABI], CA, USA) and primers F4 and R3 (Supplementary Table S6). The lower detection limit was 250 copies/ml.

### PCR Amplification, Cloning and Sequencing of HBV RT/S region

The overlapping RT/S region of HBV was amplified either by single-step PCR using primers F4 and R9 or by nested-PCR using the primer-pairs F12-R10 in 1^st^ round and F4-R9 in the 2^nd^ round (Supplementary Table 1) depending on viral load. The PCR product was either sequenced directly using Big Dye cycle sequencing kit (ABI) on automated DNA sequencer or cloned into the pJET1.2/blunt vector (Thermo Fisher Scientific, MA, USA) and transformed into *E. coli* DH5α competent cells. At least 10 clones bearing the RT/S insert were randomly selected and sequenced from each sample.

### Analysis of sequences

Sequence editing and analysis were executed using Seqscape V2.5 software. HBV/S sequences obtained in the study were further compared with representative sequences of ten HBV genotypes (A–J) available in the GenBank. Alignments were carried out using CLUSTALX software followed by the construction of phylogenetic tree by neighbor-joining method using the Kimura 2 parameter model embedded in MEGA5 software package^31^. Sequence variability in RT/S region was analyzed with the help of multiple alignment data. The HBV nucleotide and amino acid complexity of the RT/S region was evaluated for each patient by calculating normalized entropy (Sn) as described previously^9^. The quasispecies diversity, including d, dS and dN were also determined for each patient with the Kimura two-parameter method in MEGA5 program^9^.

### Homology model of HBV RT domain

The sequence of RT region of the wild-type HBV belonging to genotype D (Genbank accession no: GQ205378) was considered for three-dimensional (3D) model generation. Here, we used a previously reported^32^, manually curated HBV: HIV-1 RT domain alignment for the generation of the 3D-model. The properly curated sequence alignment matched a conserved lysine residue (K65) in HIV-1 RT domain that is essential for the ionic interaction with the γ phosphate of the incoming nucleotide and subsequent drug resistance^33^. In the final model generation, we had used HIV-1 RT domain (PDB code: 1RTD)^34^ as our guiding template crystal structure. Initially, a decoy set of 1000 models of HBV RT domain was generated using protein modeling program MODELLER^35^. The decoy set was then ranked based on MODELLER DOPE score and further top 100 models were evaluated using the PROCHECK program^36^ to select the HBV RT final model with the best stereo-chemical quality. The final model possessed 96.4% of HBV RT residues within the favored region of Ramachandran plot. CHIMERA structure visualization software^37^ was used to map the important mutations in RT that may confer resistance to LMV onto the 3D model of HBV RT domain.

### Co-mutational network analysis

Several co-occurring RT mutations were observed within the various mutant HBV strains. Networks of co-mutational sites were created using the Cytoscape software^38^. Co-mutant sites were connected via edges where edge thickness and color depict the frequency of co-mutation and the spatial distance between the mutant sites, respectively. Similarly, the distance from the mutant sites with respect to the active sites and the bound substrate (TTP) were also calculated and represented as node size within the Cytoscape network. Residues within 5 Å distance surrounding the bound substrate were considered as active site center. All spatial distances were calculated from the 3D model of HBV RT domain using in-house Perl scripts.

### Hydrophobicity plot

Hydrophobicity profiles of epitope regions of HBsAg were investigated using values normalized between 0 and 1 by Kyte–Doolittle plot using PROTSCALE program in EXPASY, a Bioinformatics Resource Portal. A sliding window of five amino acids with a step size of 1 was applied.

### Cloning of full length HBV, introduction of mutation in RT region and tenofovir susceptibility Assay

Full-length HBV genome of genotype D isolated from archival serum sample of a treatment-naïve, HBsAg- and HBeAg-positive CHB patient was amplified using primers HBVP1 and HBVP2^39^ (Supplementary Table S6), cloned into pJET1.2 vector using CloneJET PCR Cloning kit (Fermentas) and used as WT HBV. The rtH124N mutation identified in this study was introduced in WT-HBV clone (pJET-HBV-wt) by Site-Directed Mutagenesis (Agilent Technologies) with specific mutagenic oligonucleotides (rtH124N_F and rtH124N_R) (Supplementary Table S6) to generate pJET-HBV-mt (rt124N) that was confirmed by sequencing. The linear monomers of WT-HBV or mutant-HBV were released from cloning vectors by *Sap*I digestion and used for transfection in Huh7 cells. For antiviral assay, Tenofovir, GS-1278 was provided by Gilead Sciences (Foster City, CA, USA) and six-well culture dishes were seeded with 7.5 × 10^5^ Huh7 cells/well and transfected with 2 μg of linear monomers of WT-HBV or mutant–HBV by using Lipofectamine 2000 (Invitrogen), together with pRL-CMV *Renilla Luciferase* Reporter Vector (Promega), which served as transfection normalization control. Six hours after transfection, cells were washed with PBS and the culture medium was replaced with media containing tenofovir at concentrations from 0 to 50 μM. The cells were again fed with fresh medium containing appropriate concentration of the drug after seventy-two hours and harvested at day 5 post-transfection. The cells were lysed with lysis buffer (50 mM Tris–HCl pH 8.0; 1 mM EDTA; 1% Nonidet P40), HBV DNA was isolated from intracellular core particles as described previously^40^ and luciferase assay was also performed using a Luciferase Reporter Assay kit (Promega). HBV DNA from core particles was quantified by real-time PCR and results were normalized to *Renilla luciferase* readings. The 50% inhibitory concentrations (IC_50_) of the drug for both WT and mutant HBV was determined by a 50% decrease in the amount of intracellular viral DNA detected in treated cells at the end of the treatment compared with untreated cells^41^ and the fold of resistance towards tenofovir was determined by dividing mutant IC_50_ by WT IC_50_.

### Statistical Analysis

Data were expressed as mean ± standard deviation (SD) or median (range) as relevant. Statistical analysis was performed using SPSS 18.0 (SPSS Inc., Chicago, IL). Continuous variables with normal and skewed distributions were compared between groups using the Student t test and the Mann-Whitney test, respectively. Categorical variables were tested using the Fisher’s exact test. The Benjamini-Hochberg correction with a false-discovery rate of 5% was applied to all p-values, to control the proportion of false-positive associations resulting from multiple testing^42^. Statistical significance was denoted by p < 0.05.

## Additional Information

**How to cite this article:** Banerjee, P. *et al*. HBV quasispecies composition in Lamivudine-failed chronic hepatitis B patients and its influence on virological response to Tenofovir-based rescue therapy. *Sci. Rep.* **7**, 44742; doi: 10.1038/srep44742 (2017).

**Publisher's note:** Springer Nature remains neutral with regard to jurisdictional claims in published maps and institutional affiliations.

## Supplementary Material

Supplementary Data

## Figures and Tables

**Table 1:** Frequency of amino acid substitutions in HBV/RT in the viral quasi species pool from LMV-failed (n = 16) and treatment-naïve (n = 14) CHB patients.

Amino acid substitutions in HBV/RT		Frequency (%) in HBV clonal population in LMV-failed CHB patients (total no of clones = 160)	Frequency (%) in HBV clonal population in treatment naïve-CHB patients (total no of clones = 140)	*p*-value (unadjusted)	Benjamini- Hochberg threshold
**rtM204V**	Known LMV- resistant mutations	26.3	0	2.05E-13*	0.000363
**rtM204I**		15.6	0	1.12E-07*	0.001962
**rtS202G**		17.5	0	1.12E-08*	0.00116
**rtL80I**		15.6	0	1.12E-07*	0.001962
**rtL180M**		26.3	0	2.05E-13*	0.000363
**rtN53D**	Novel amino acid substitutions	39.4	20	3.00E-04*	0.002326
rtY54H		43.1	27.1	5.30E-03	0.002616
**rtL91I**		8.8	0	1.00E-04*	0.00218
**rtI121L**		24.4	0	2.19E-12*	0.000436
**rtF**/**H122L**		42.5	14.3	6.56E-08*	0.001599
**rtH124N**		48.8	12.9	1.88E-11*	0.000727
**rtQ130P**		41.9	11.4	2.86E-09*	0.001017
**rtN131D**		50.6	15.7	1.27E-10*	0.000872
**rtA219S**		94.4	82.1	9.00E-04*	0.002471
**rtH248N**		78.8	56.4	4.00E-05*	0.002035
rtS256C		89.4	80.7	4.88E-2	0.002762
**rtR266I**		90.6	64.3	4.39E-08*	0.001453
**rtH**/**L267Q**		92.5	67.1	2.48E-08*	0.001308
**rtV278I**		50	12.9	4.06E-12*	0.000581

^*^Significant after Benjamini-Hochberg correction; HBV/rt variants with significantly different frequency distribution after Benjamini-Hochberg correction are shown in bold.

**Table 2:** Frequency of amino acid substitutions in HBV/S in the viral quasi species pool from LMV-failed (n = 16) and treatment-naïve (n = 14) CHB patients.

Amino acid substitutions in HBV/S	Location in Non-epitope/Epitope region)	Frequency (%) in HBV clonal population in LMV-failed CHB patients (total no of clones = 160)	Frequency (%) in HBV clonal population in treatment-naïve CHB patients (total no of clones = 140)	*p*-value (unadjusted)	Benjamini- Hochberg threshold
sN3S	non-epitope	22.5	12.1	2.26E-02	0.004535
sI4T	non-epitope	23.8	12.1	1.09E-02	0.003319
sS45A	B-, Th-, Tc-cell epitope	26.9	14.3	1.02E-02	0.003097
sV47T	B-, Th-, Tc-cell epitope	20	8.6	5.40E-03	0.002876
sL49P	B-, Th-, Tc-cell epitope	23.1	12.1	1.58E-02	0.004093
sS53L	non-epitope	23.1	12.1	1.58E-02	0.004093
sA/S113T	non-epitope	22.5	12.1	2.26E-02	0.004535
**sT114S**	non-epitope	85.6	69.3	7.68E-04*	0.002323
sT118V	B-cell epitope	30.6	17.9	1.10E-02	0.00354
**sT126I**	B-, Th-cell epitope	19.4	5.0	1.90E-04*	0.00177
**sP127T**	B-, Th-cell epitope	61.3	34.3	3.45E-06*	0.001106
sA128V	B-, Th- cell epitope	32.5	17.9	5.21E-03	0.002655
**sN131T**	B-, Th- cell epitope	90.6	69.3	3.68E-06*	0.001327
**sN143S**	B-, Th- cell epitope	85.6	69.3	7.68E-04*	0.002323
**sK160R**	B-cell epitope	25.6	12.1	3.36E-03	0.002434
**sY**/**S161F**	B-cell epitope	87.5	62.9	7.34E-07*	0.000664
**sA194V**	B-, Tc- cell epitope	85	62.1	9.64E-06*	0.001549
**sI195M**	B-cell epitope	25	0	9.98E-13*	0.000221
**sW196L**	B-cell epitope	14.4	0	2.46E-07*	0.000442
**sR210N**	Tc epitope	25	5.7	3.19E-06*	0.000885

Abbreviation- Tc, cytotoxic T cell epitope; Th, helper T cell epitope.

^*^Significant after Benjamini-Hochberg correction; HBV/S variants with significantly different frequency distribution after Benjamini-Hochberg correction are shown in bold.

**Table 3:** Baseline frequency of amino acid substitutions in RT and S regions of HBV quasispecies from LMV-failed CHB patients who responded differentially to add-on TDF therapy.

Amino acid substitutions	Baseline frequency (%) in HBV clones from six TDF responders (total no of clones = 60)	Baseline frequency (%) in HBV clones from four TDF nonresponders (total no of clones = 40)	*p*-value (unadjusted)	Benjamini- Hochberg threshold
**HBV**/**RT**				
rtN53D	27.5	51.7	1.30E-02	0.001163
rtY54H	60	25	5.00E-04	0.000436
rtL91I	6.7	20	4.62E-02	0.001744
rtT118N	83.3	100	4.40E-03	0.001017
rtI121L	3.3	25	1.60E-03	0.000727
rtF/H122L	25	47.5	1.76E-02	0.001526
**rtH124N**	25	72.5	4.03E-06*	0.000145
rtQ130P	25	47.5	1.76E-02	0.001526
rtS256C	76.7	100	4.00E-04	0.000291
rtR266I	80	100	1.30E-03	0.000581
rtH/L267Q	75	97.5	1.70E-03	0.000872
rtV278I	23.3	45	2.94E-02	0.001599
**HBV**/**S**				
**sS45A**	21.7	75	1.45E-07*	0.001106
sP46T	53.4	75	2.31E-02	0.001880531
sI68T/A	53.4	75	2.31E-02	0.001880531
**sA**/**S113T**	10	75	4.05E-11*	0.000442
**sT118V**	21.7	90	6.69E-12*	0.000221
**sT125M**	11.7	75	1.07E-10*	0.000664
**sA128V**	31.7	82.5	5.85E-07*	0.001327
**sR210N**	16.7	75	5.79E-09*	0.000885

^*^Significant after Benjamini-Hochberg correction; variants with significantly different frequency distribution after Benjamini-Hochberg correction are shown in bold.

**Table 4:** Frequency of amino acid substitutions in RT and S regions of HBV quasispecies from LMV-failed CHB patients who responded differentially after 24 week TDF add-on therapy.

Amino acid substitutions in HBV RT and S region	Baseline frequency (%) in HBV clones	Frequency (%) in HBV clones after 24 week TDF therapy	*p*-value (unadjusted)	Benjamini-Hochberg threshold
**TDF Responders** (total no of HBV clones = 60)				
HBV/RT				
**rtL80I**	25	0	2.25E-05*	0.000509
**rtM204I**	25	0	2.25E-05*	0.000509
rtN53D	27.5	11.7	3.90E-02	0.001017
**rtY54H**	60	16.7	1.69E-06*	0.000145
rtH124N	25	8.3	2.60E-02	0.000872
rtN131D	45	16.7	1.40E-03	0.000581
rtH248N	60	33.3	5.80E-03	0.000727
HBV/S				
**sS45A**	21.7	0	1.20E-04*	0.001217
**sP46T**	53.3	16.7	4.40E-05*	0.000774
**sI68T**/**A**	53.3	16.7	4.40E-05*	0.000774
sA/S113T	10	0	2.74E-02	0.00177
**sT118V**	21.7	0	1.20E-04*	0.001217
sT125M	11.7	0	1.29E-02	0.001549
**sW196L**	25	0	2.25E-05*	0.000221
**sR210N**	16.7	0	1.29E-03*	0.001327
**TDF Non Responders** (total no of HBV clones = 40)				
HBV/RT				
rtL80I	25	0	1.03E-03	0.000363372
rtM204I	25	0	1.03E-03	0.000363372
HBV/S				
sW196L	25	0	1.00E-03	0.000221239

^*^Significant after Benjamini-Hochberg correction; variants with significantly different frequency distribution after Benjamini-Hochberg correction are shown in bold.

**Table 5:** Frequency of amino acid substitutions in RT and S regions of HBV quasispecies from LMV-failed CHB patients who responded differentially after 48 week TDF add-on therapy.

Amino acid substitutions in HBV RT and S region	Baseline frequency (%) in HBV clones	Frequency (%) in HBV clones after 48 week TDF therapy	*p*-value (unadjusted)	Benjamini-Hochberg threshold
**TDF Responders** (total no of HBV clones = 60)				
HBV/RT				
**rtL80I**	25	1.7	2.20E-04*	0.000363
**rtM204I**	25	1.7	2.20E-04*	0.000363
rtY54H	60	33.3	5.80E-03	0.000872
rtN131D	45	18.3	2.90E-03	0.000581
rtA219S	90	100	2.70E-02	0.001163
rtH248N	60	33.3	5.80E-03	0.000727
rtS256C	76.7	93.3	1.90E-02	0.001017
**rtV278I**	23.3	56.7	3.50E-04*	0.000436
HBV/S				
**sS45A**	21.7	0	1.18E-04*	0.000774
sP46T	53.3	25	2.59E-03	0.001659
sI68T/A	53.3	25	2.59E-03	0.001659
sA/S113T	10	0	2.74E-02	0.001991
**sT118V**	21.7	0	1.18E-04*	0.000774
sT125M	11.7	0	1.29E-02	0.00177
**sA128V**	31.7	0	7.01E-07*	0.000221
**sW196L**	25	1.7	2.15E-04*	0.000885
sR210N	16.7	0	1.29E-03	0.001106
**TDF Non Responders** (total no of HBV clones = 40)				
HBV/RT				
rtL80I	25	0	1.03E-03	0.000363
rtM204I	25	0	1.03E-03	0.000363
rtL91I	20	0	5.30E-03	0.000581
rtI121L	25	0	1.03E-03	0.000436
rtH248N	75	50	3.70E-02	0.000727
HBV/S				
sA128V	82.5	100	1.17E-02	0.000442478
sW196L	25	0	1.00E-03	0.000221239

^*^Significant after Benjamini-Hochberg correction; variants with significantly different frequency distribution after Benjamini-Hochberg correction are shown in bold.

## References

[b1] Zoulim F. & Locarnini S. Hepatitis B virus resistance to nucleos(t)ide analogues. Gastroenterology 137, 1593–1608 (2009). 19737565 10.1053/j.gastro.2009.08.063

[b2] Rehermann B. & Bertoletti A. Immunological aspects of antiviral therapy of chronic hepatitis B virus and hepatitis C virus infections. Hepatology 61, 712–721 (2015). 25048716 10.1002/hep.27323 PMC4575407

[b3] Ayoub W. S. & Keeffe E. B. Review article: current antiviral therapy of chronic hepatitis B. Aliment. Pharmacol. Ther. 34, 1145–1158 (2011). 21978243 10.1111/j.1365-2036.2011.04869.x

[b4] Deng X. L., Li Q. L. & Guo J. J. Dynamics of lamivudine-resistant hepatitis B virus strains in patients with entecavir rescue therapy. Virus Genes 47, 1–9 (2013). 23616174 10.1007/s11262-013-0915-1

[b5] European Association For The Study Of The Liver. EASL clinical practice guidelines: Management of chronic hepatitis B virus infection. J. Hepatol. 57, 167–185 (2012). 22436845 10.1016/j.jhep.2012.02.010

[b6] Rodriguez-Frías F. . Ultra-deep pyrosequencing detects conserved genomic sites and quantifies linkage of drug-resistant amino acid changes in the hepatitis B virus genome. PLoS One 7, e37874 (2012). 22666402 10.1371/journal.pone.0037874 PMC3364280

[b7] Mirandola S. . Genotype-specific mutations in the polymerase gene of hepatitis B virus potentially associated with resistance to oral antiviral therapy. Antiviral Res. 96, 422–429 (2012). 23026293 10.1016/j.antiviral.2012.09.014

[b8] Lee Y. B. . Tenofovir monotherapy versus tenofovir plus lamivudine or telbivudine combination therapy in treatment of lamivudine-resistant chronic hepatitis B. Antimicrob. Agents Chemother. 59, 972–978 (2015). 25421484 10.1128/AAC.04454-14 PMC4335865

[b9] Chen L., Zhang Q., Yu D. M., Wan M. B. & Zhang X. X. Early changes of hepatitis B virus quasispecies during lamivudine treatment and the correlation with antiviral efficacy. J. Hepatol. 50, 895–905 (2009). 19304333 10.1016/j.jhep.2008.12.018

[b10] Howard C. R. & Allison L. M. Hepatitis B surface antigen variation and protective immunity. Intervirology 38, 35–40 (1995). 8666522 10.1159/000150412

[b11] Mondal R. K. . Immune driven adaptation of hepatitis B virus genotype D involves preferential alteration in B cell epitopes and replicative attenuation–an insight from human immunodeficiency virus/hepatitis B virus coinfection. Clin. Microbiol. Infect. 21, 710. e11–20 (2015). 25882358 10.1016/j.cmi.2015.03.004

[b12] Wakil S. M. . Prevalence and profile of mutations associated with lamivudine therapy in Indian patients with chronic hepatitis B in the surface and polymerase genes of hepatitis B virus. J. Med. Virol. 68, 311–318 (2002). 12226816 10.1002/jmv.10205

[b13] Ismail A. M. . Lamivudine monotherapy in chronic hepatitis B patients from the Indian subcontinent: antiviral resistance mutations and predictive factors of treatment response. Mol. Diagn. Ther. 18, 63–71 (2014). 24030850 10.1007/s40291-013-0054-3

[b14] Ciancio A. . Identification of HBV DNA sequences that are predictive of response to lamivudine therapy. Hepatology 39, 64–73 (2004). 14752824 10.1002/hep.20019

[b15] Wong D. K. . Effect of hepatitis B virus reverse transcriptase variations on entecavir treatment response. J. Infect. Dis. 210, 701–707 (2014). 24610871 10.1093/infdis/jiu133

[b16] Torresi J. . Reduced antigenicity of the hepatitis B virus HBsAg protein arising as a consequence of sequence changes in the overlapping polymerase gene that are selected by lamivudine therapy. Virology 293, 305–313 (2002). 11886250 10.1006/viro.2001.1246

[b17] Ghany M. G. . Hepatitis B virus S mutants in liver transplant recipients who were reinfected despite hepatitis B immune globulin prophylaxis. Hepatology 27, 213–222 (1998). 9425940 10.1002/hep.510270133

[b18] Chisari F. V. & Ferrari C. Hepatitis B virus immunopathogenesis. Annu. Rev. Immunol. 13, 29–60 (1995). 7612225 10.1146/annurev.iy.13.040195.000333

[b19] Solmone M. . Slow response to entecavir treatment in treatment-naive HBV patients is conditioned by immune response rather than by the presence or selection of refractory variants. Antivir. Ther. 19, 201–209 (2014). 24275042 10.3851/IMP2700

[b20] Schmutz G. . Combination of TDF and LMV versus TDF after LMV failure for therapy of hepatitis B in HIV-coinfection. AIDS 20, 1951–1954 (2006). 16988516 10.1097/01.aids.0000247116.89455.5d

[b21] Lee S. . Tenofovir versus tenofovir plus entecavir for chronic hepatitis B with lamivudine resistance and entecavir resistance. J. Viral Hepat. 24, 141–147 (2017). 27766731 10.1111/jvh.12623

[b22] Kumada H., Koike K., Suyama K., Ito H., Itoh H. & Sugiura W. Efficacy and safety of tenofovir disoproxil fumarate rescue therapy for chronic hepatitis B patients who failed other nucleos(t)ide analogs. Hepatol. Res., doi: 10.1111/hepr.12842 (2016). 27862721

[b23] De Francesco M. A. . Clinical course of chronic hepatitis B patients receiving nucleos(t)ide analogues after virological breakthrough during monotherapy with lamivudine. New Microbiol. 38, 29–37 (2015). 25742145

[b24] Liu F. . Evolutionary patterns of hepatitis B virus quasispecies under different selective pressures: correlation with antiviral efficacy. Gut 60, 1269–1277 (2011). 21292683 10.1136/gut.2010.226225

[b25] Lavocat F. . Similar evolution of hepatitis B virus quasispecies in patients with incomplete adefovir response receiving tenofovir/emtricitabine combination or tenofovir monotherapy. J. Hepatol. 59, 684–695 (2013). 23742912 10.1016/j.jhep.2013.05.038

[b26] Chin R. . *In vitro* susceptibilities of wild-type or drug-resistant hepatitis B virus to (-)-beta-D-2,6-diaminopurine dioxolane and 2′-fluoro-5-methyl-beta-L-arabinofuranosyluracil. Antimicrob. Agents Chemother. 45, 2495–2501 (2001). 11502520 10.1128/AAC.45.9.2495-2501.2001 PMC90683

[b27] Lada O. . *In vitro* susceptibility of lamivudine-resistant hepatitis B virus to adefovir and tenofovir. Antivir. Ther. 9, 353–363 (2004). 15259898

[b28] Delaney W. E. 4th. . Intracellular metabolism and *in vitro* activity of tenofovir against hepatitis B virus. Antimicrob. Agents Chemother. 50, 2471–2477 (2006). 16801428 10.1128/AAC.00138-06 PMC1489769

[b29] Haas G. . Dynamics of viral variants in HIV-1 Nef and specific cytotoxic T lymphocytes *in vivo*. J. Immunol. 157, 4212–4221 (1996). 8892659

[b30] Nowak M. A. . Antigenic oscillations and shifting immunodominance in HIV-1 infections. Nature 375, 606–611 (1995). 7791879 10.1038/375606a0

[b31] Banerjee P. . A rare HBV subgenotype D4 with unique genomic signatures identified in north-eastern India–an emerging clinical challenge? PLoS One 9, e109425 (2014). 25295865 10.1371/journal.pone.0109425 PMC4190083

[b32] Daga P. R., Duan J. & Doerksen R. J. Computational model of hepatitis B virus DNA polymerase: molecular dynamics and docking to understand resistant mutations. Protein Sci. 19, 796–807 (2010). 20162615 10.1002/pro.359 PMC2867019

[b33] Gu Z. . K65R mutation of human immunodeficiency virus type 1 reverse transcriptase encodes cross-resistance to 9-(2-phosphonylmethoxyethyl) adenine. Antimicrob. Agents Chemother. 39, 1888–1891 (1995). 7486942 10.1128/aac.39.8.1888 PMC162849

[b34] Huang H., Chopra R., Verdine G. L. & Harrison S. C. Structure of a covalently trapped catalytic complex of HIV-1 reverse transcriptase: implications for drug resistance. Science 282, 1669–1675 (1998). 9831551 10.1126/science.282.5394.1669

[b35] Eswar N. . Comparative protein structure modeling using MODELLER. Curr. Protoc. Protein Sci.Chapter 2: Unit 2 9 (2007). 10.1002/0471140864.ps0209s50 18429317

[b36] Laskowski R. A., MacArthur M. W., Moss D. S. & Thornton J. M. PROCHECK - a program to check the stereochemical quality of protein structures. J. App. Cryst. 26, 283–291 (1993).

[b37] Pettersen E. F. . UCSF Chimera–a visualization system for exploratory research and analysis. J. Comput. Chem. 25, 1605–1612 (2004). 15264254 10.1002/jcc.20084

[b38] Shannon P. . Cytoscape: a software environment for integrated models of biomolecular interaction networks. Genome Res. 13, 2498–2504 (2003). 14597658 10.1101/gr.1239303 PMC403769

[b39] Günther S., Li B. C., Miska S., Krüger D. H., Meisel H. & Will H. A novel method for efficient amplification of whole hepatitis B virus genomes permits rapid functional analysis and reveals deletion mutants in immunosuppressed patients. J. Virol. 69, 5437–44 (1995). 7636989 10.1128/jvi.69.9.5437-5444.1995 PMC189390

[b40] Durantel D. . A new strategy for studying *in vitro* the drug susceptibility of clinical isolates of human hepatitis B virus. Hepatology 40, 855–864 (2004). 15382118 10.1002/hep.20388

[b41] Seignères B., Pichoud C., Martin P., Furman P., Trépo C. & Zoulim F. Inhibitory activity of dioxolane purine analogs on wild-type and lamivudine-resistant mutants of hepadnaviruses. Hepatology 36, 710–722 (2002). 12198665 10.1053/jhep.2002.35070

[b42] Benjamini Y. & Hochberg Y. Controlling the false discovery rate-a practical and powerful approach to multiple testing. J. Roy. Stat. Soc. B. 57, 289–300 (1995).

