# Supplementary material for: HBV quasispecies composition in Lamivudine-failed chronic hepatitis B patients and its influence on virological response to Tenofovir-based rescue therapy
Source: Sci Rep. 2017 Mar 17;7:44742. doi: 10.1038/srep44742 (PMC5356183; doi:10.1038/srep44742)
Supplement: Supplementary Data [file srep44742-s1.pdf]

HBV quasispecies composition in Lamivudine-failed chronic hepatitis B patients and its influence on virological response to Tenofovir-based rescue therapy

Priyanka Banerjee, Abhijit Chakraborty, Rajiv Kumar Mondal, Mousumi Khatun, Somenath Datta, Kausik Das, Pratap Pandit, Souvik Mukherjee, Soma Banerjee, Saurabh Ghosh, Saikat Chakrabarti, Abhijit Chowdhury and Simanti Datta

Supplementary Table S1. Clinical, demographic and biochemical data of LMV-failed and treatment-naïve CHB patients infected with HBV of genotype D

	LMV-failed CHB patients (n=16)	Treatment-naïve CHB patients (n=14)	*p*-value
Age (Year), median (range)	46.5 (20-63)	46 (10-67)	>0.05
Sex, M:F	13:3	13:1	>0.05
ALT (IU/L), median (range)	66 (46-93)	74 (47-174)	>0.05
AST (IU/L), median (range)	45 (33-127)	60 (44-181)	>0.05
HBV DNA (Log_{10} copies/mL), mean±SD	5.839 ± 0.4046	6.654 ± 0.5043	>0.05
HBeAg (Positive: Negative)	12:4	7:7	>0.05
Total bilirubin, mean±SD	0.58±0.05	1.22±0.74	>0.05

Abbreviations: ALT, alanine aminotransferase; AST, aspartate aminotransferase; IU, international unit; HBeAg, Hepatitis B e-antigen

Supplementary Table S2. Frequently observed co-mutation sites in HBV/RT

Site 1 in RT	Site 2 In RT	Frequency	Distance between the sites (Å)	Minimum Distance from the active center (Site 1)	Minimum Distance from the active center (Site 2)
248	278	50	22.27	5.56	22.45
122	124	42.5	4.13	19.06	25.23
124	248	42.5	47.86	25.23	5.56
122	130	41.875	22.90	19.06	26.92
124	130	41.875	17.41	25.23	26.92
130	248	41.875	50.04	26.92	5.56
122	278	40.625	44.31	19.06	22.45
124	278	40.625	51.91	19.06	22.45
130	278	40	60.26	26.92	22.45

Supplementary Table S3. Clinical, demographic and biochemical data of LMV-failed patients responding differentially to TDF add-on therapy

Baseline parameters	Responders to TDF add-on therapy (n=6)	Nonresponders to TDF add-on therapy (n=4)
Age (Year), median (range)	34.5 (15-62)	49.50 (27-55)
Sex, M:F	5:1	3:1
ALT (IU/L), median (range)	54 (41-67)	72 (65-93)
HBV DNA (Log_{10} copies/mL), mean±SD	6.526 ± 0.4680	3.818 ± 0.3460
HBeAg (Pos:Neg)	4:2	2:2

Abbreviations: ALT, alanine aminotransferase; IU, international unit

Supplementary Table S4. Baseline HBV quasispecies complexity and diversity in RT/S region in LMV-failed CHB patients showing differential response to TDF add-on therapy

	TDF Responders (n=6) (total no of HBV clones analyzed=60)	TDF Nonresponders (n=4) (total no of HBV clones analyzed=40)	*p*-value
Quasispecies complexity (nucleotide level) (median, range)	0.7850 (0.5700-1.000)	0.9300 (0.7500-1.000)	>0.05
Quasispecies complexity (amino acid level) (median, range)	0.7500 (0.5500-0.8700)	0.9300 (0.9300-0.9400)	**<0.05**
d (nucleotide level) (median, range)	0.0065 (0.0010-0.0110)	0.0080 (0.0030-0.0140)	>0.05
d (amino acid level) (median, range)	0.0115 (0.0030-0.0300)	0.0125 (0.0090-0.0240)	>0.05
dS (substitution/site) (median, range)	0.0075 (0.0010-0.0220)	0.0095 (0.0010-0.0280)	>0.05
dN (substitution/site) (median, range)	0.0055 (0.0010-0.0120)	0.0075 (0.0040-0.0200)	>0.05

Abbreviations: d, mean genetic distance; dS, number of synonymous substitutions per synonymous site; dN, number of nonsynonymous substitutions per nonsynonymous site

Supplementary Table S5. Evolution of HBV quasi species heterogeneity among the LMV-failed patients during TDF add-on therapy

Patients	0 day (Baseline)	24 week TDF therapy	48 week TDF therapy	*p*-value (0 day vs 24 week)	*p*-value (0 day vs 48 week)
**TDF Responders** (total no of HBV clones analyzed=60)					
Quasispecies complexity (nucleotide level) (median, range)	0.7850 (0.5700-1.000)	0.3900 (0.2500-0.5600)	0.2900 (0.2900-0.3700)	**<0.05***	**<0.05***
Quasispecies complexity (amino acid level) (median, range)	0.7500 (0.5500-0.8700)	0.3600 (0.2700-0.4500)	0.2900 (0.2000-0.3900)	**<0.05***	**<0.05***
d (nucleotide level) (median, range)	0.0065 (0.0010-0.0110)	0.0035 (0.0010-0.0045)	0.0020 (0.0010-0.0040)	**<0.05***	**<0.05***
d (amino acid level) (median, range)	0.0115 (0.0030-0.0300)	0.0060 (0.0025-0.0110)	0.0030 (0.0010-0.0100)	**<0.05***	**<0.05***
dS (substitution/site) (median, range)	0.0075 (0.0010-0.0220)	0.0030 (0.0010-0.0060)	0.0015 (0.0010-0.0020)	**<0.05***	**<0.05***
dN (substitution/site) (median, range)	0.0055 (0.0010-0.0120)	0.0029 (0.0008-0.0045)	0.0015 (0.0010-0.0030)	**<0.05***	**<0.05***
**TDF Nonresponders** (total no of HBV clones analyzed=40)					
Quasispecies complexity (nucleotide level) (median, range)	0.9300 (0.7500-1.000)	0.6800 (0.5500-0.7200)	0.3900 (0.2900-0.4600)	>0.05	**<0.05***
Quasispecies complexity (amino acid level) (median, range)	0.9300 (0.9300-0.9400)	0.6500 (0.3900-0.8800)	0.2950 (0.2500-0.5700)	>0.05	**<0.05***
d (nucleotide level) (median, range)	0.0080 (0.0030-0.0140)	0.0060 (0.0010-0.0090)	0.0045 (0.0008-0.0080)	>0.05	>0.05
d (amino acid level) (median, range)	0.0125 (0.0090-0.0240)	0.0090 (0.0065-0.0180)	0.0075 (0.0040-0.0150)	>0.05	>0.05
dS (substitution/site) (median, range)	0.0095 (0.0010-0.0280)	0.0070 (0.0015-0.0200)	0.0060 (0.0010-0.0200)	>0.05	>0.05
dN (substitution/site) (median, range)	0.0075 (0.0040-0.0200)	0.0060 (0.0020-0.0120)	0.0045 (0.0010-0.0140)	>0.05	>0.05

Abbreviations: d, mean genetic distance; dS, number of synonymous substitutions per synonymous site; dN, number of nonsynonymous substitutions per nonsynonymous site

Supplementary Table S6: List of primers used in the study

Primer Name	Primer Sequences (5'-3')
F4	CTCAGGCCATGCAGTGGAA
R3	AACTGGAGCCACCAGCAG
R9	TAGGAGTTCCGCAGTATGGA
F12	TCTCAATCGCCGCGTCGCAG
R10	CAGCCTCCTAGTACAAAGAC
#HBVP1	CCGGAAAGCTTGAGCTCTTCTTTTTCACCTCTGCCTAATCA
#HBVP2	CGGAAAGCTTGAGCTCTTCAAAAAGTTGCATGGTGCTGG
rtH124N_F	CTAATTCCAGGATCTTCGACAACCAGCACGGGACCATGC
rtH124N_R	GCATGGTCCCGTGCTGGTGGTCGAAGATCCTGGAATTAG

#Günther S *et al.* J Virol 1995; 69: 5437-5444

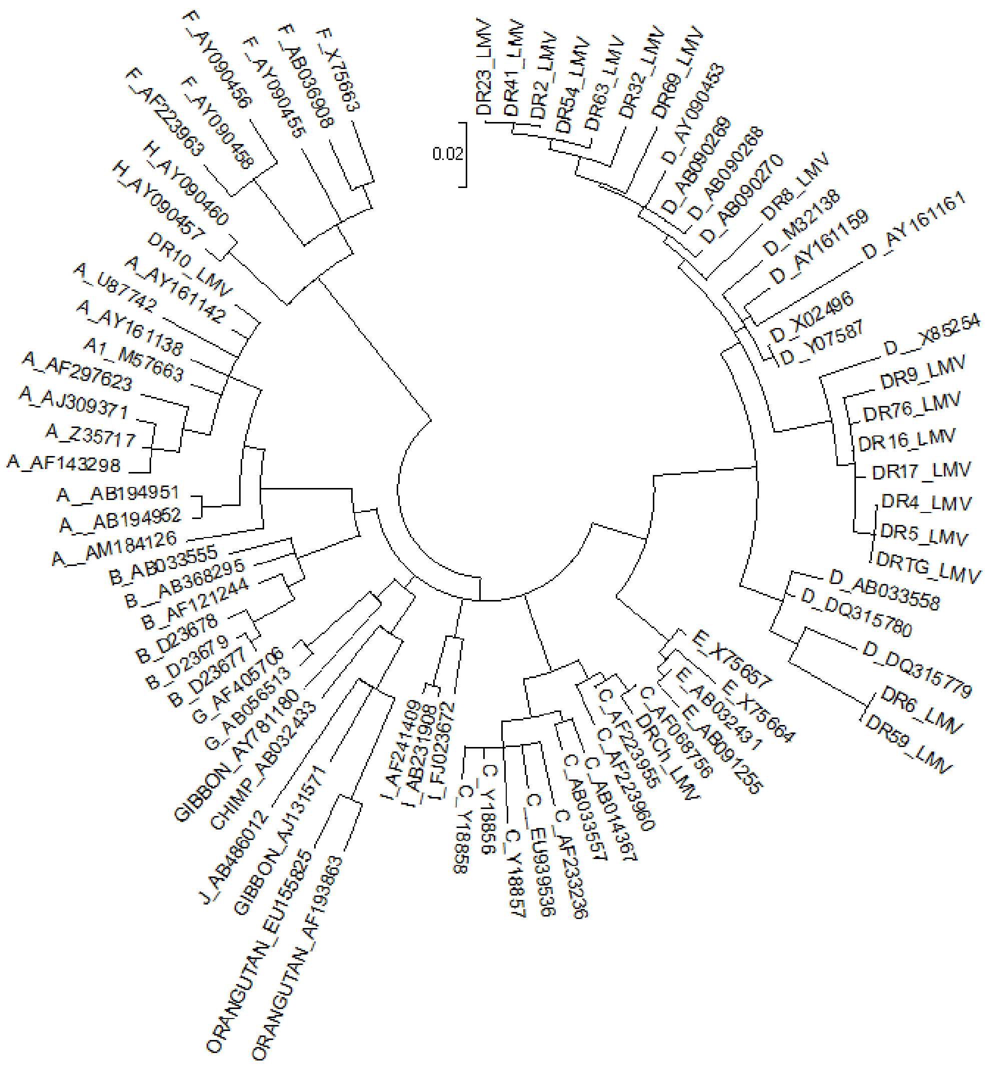

Supplementary Fig. S1A: Phylogenetic analysis of the HBV/S sequences of 18 LMV-failed CHB patients along with reference sequences of HBV/S belonging to genotypes (A-J) retrieved from GenBank, including sequences from nonhuman primates. HBV sequences from GenBank are indicated by their genotypes followed by accession numbers. The sequences determined in the study are given by the isolate number initiated with DR and followed by the clinical status of the patients (LMV-failed, LMV) from whom it was derived. The phylogenetic tree was built using the Jukes Cantor and neighbour joining method by MEGA software version6 and bootstrap resampling and reconstruction were carried out 5000 times. Out of 18 LMV treated patients, 16 patients (88.9%) were infected with the HBV of genotype D, 1 patient (5.6%) carried HBV of genotype A and 1 patient (5.6%) had HBV belonging to genotype C.

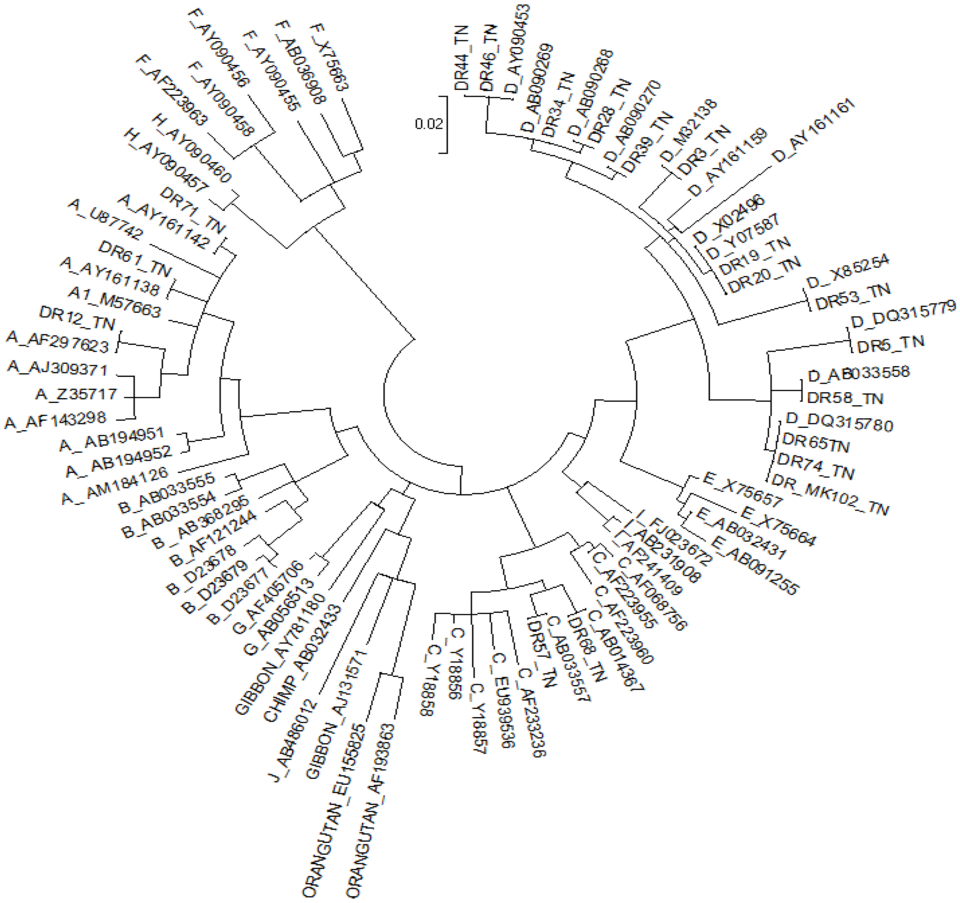

Supplementary Fig. S1B: Phylogenetic analysis of the HBV/S sequences of 19 treatment-naïve CHB patients along with reference sequences of HBV/S belonging to genotypes (A-J) retrieved from GenBank, including sequences from nonhuman primates. HBV sequences from GenBank are indicated by their genotypes followed by accession numbers. The sequences determined in the study are given by the isolate number initiated with DR and followed by the clinical status of the patients (treatment-naïve, TN) from whom it was derived. The phylogenetic tree was built using the Jukes Cantor and neighbour joining method by MEGA software version6 and bootstrap resampling and reconstruction were carried out 5000 times. Among the 19 treatment-naïve CHB patients, 14 patients (73.7%) harbored HBV/D, 3 patients (15.8%) carried HBV/A and 2 (10.5%) were infected with HBV/C.

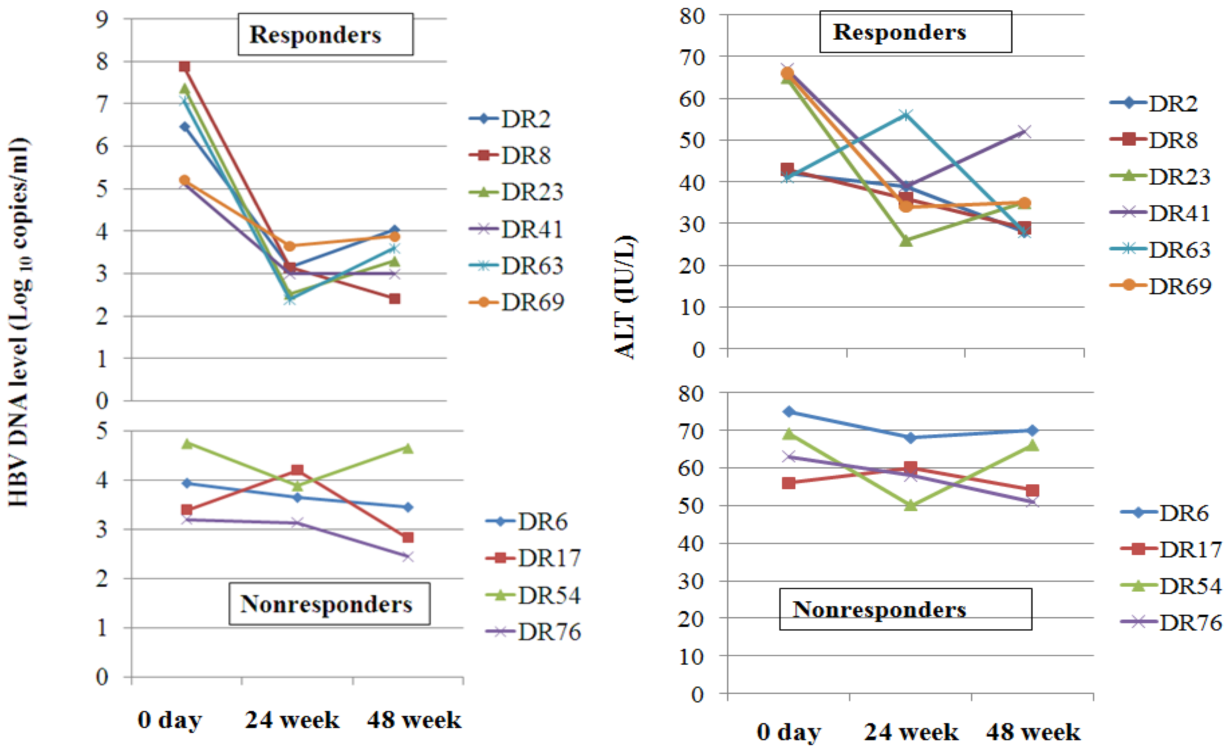

Supplementary Fig. S2: Serum HBV DNA and ALT kinetics at baseline (0 day) and after 24 week and 48 week LMV+ TDF add-on therapy in LMV-failed CHB patients . Six out of 10 LMV-failed patients responded to TDF therapy while four patients were non-responders.

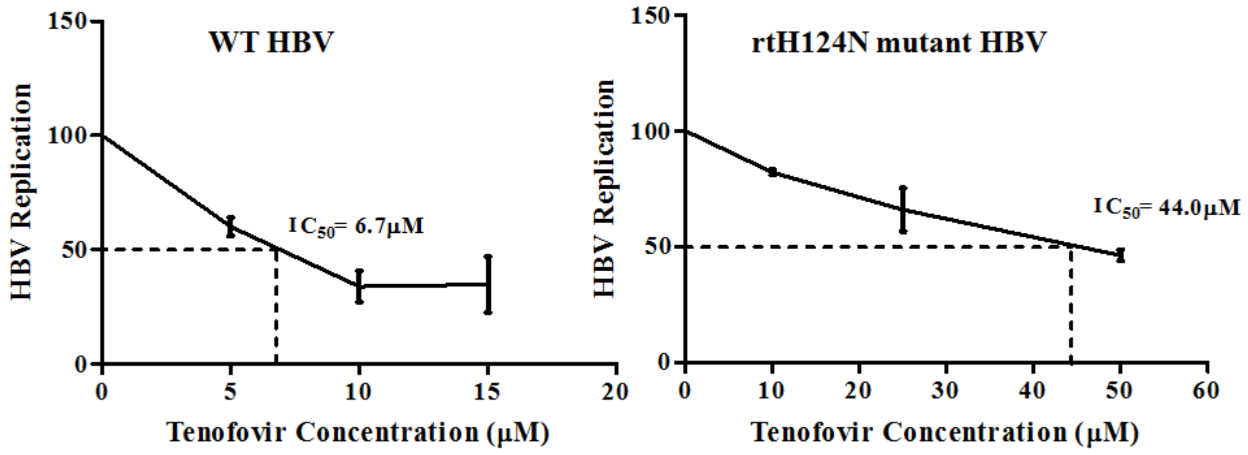

Supplementary Fig. S3: Susceptibility of wild type (WT) HBV and rtH124N mutant HBV to Tenofovir. Huh7 hepatoma cells were transiently transfected with linear monomers of wild-type (WT) HBV or rtH124N mutant HBV together with pRL-CMV *Renilla Luciferase* Reporter Vector, which served as transfection normalization control. Tenofovir, GS-1278 was added to the culture medium at different concentrations as indicated. HBV DNA from core particles was quantified as a measure of HBV replication by real-time PCR at day 5 post-transfection and results were normalized to *Renilla luciferase* readings. The 50% inhibitory concentrations (IC_{50}) of the drug for both WT and mutant HBV was determined by a 50% decrease in the amount of intracellular viral DNA detected in treated cells at the end of the treatment compared with untreated cells. Means and standard deviations are based on three independent experiments.
